# Gut Catalase-Positive Bacteria Cross-Protect Adjacent Bifidobacteria from Oxidative Stress

**DOI:** 10.1264/jsme2.ME15025

**Published:** 2015-06-04

**Authors:** Eva Rodríguez, Ángela Peirotén, José María Landete, Margarita Medina, Juan Luis Arqués

**Affiliations:** 1 Dpto. de Tecnología de Alimentos, INIA, Carretera de La Coruña km 7, 28040 Madrid, Spain

**Keywords:** *Bifidobacterium*, oxidative stress, catalase, cross-protection, infant gut

## Abstract

Bifidobacteria isolated from infant gut and breast milk exhibited different abilities to grow under microaerobic conditions, alone or in the presence of added catalase. In the present study, we demonstrated that some *Bifidobacterium* strains unable to grow under microaerobic conditions were cross-protected on solid media from oxidative stress by adjacent colonies of gut catalase-positive *Staphylococcus epidermidis* or *Escherichia coli*, but not by a catalase-deficient *E. coli*. The results of this study support the possible contribution of catalase-positive bacteria to the establishment of certain bifidobacteria in non-anaerobic human niches of the infant gastrointestinal tract or mammary gland.

Colonization of the gut is described as a complex process influenced by microbial-host interactions as well as by external and internal factors. Climax intestinal microbiota in newborns is attained in successive stages. The intestinal and vaginal microbiota and breast milk of the mother are the main sources of bacteria for the neonate during the first days of life (6, 14, 15). The first colonizers of the gastrointestinal tract are bacteria that are tolerant to oxygen such as *Escherichia coli* and other *Enterobacteriaceae*, enterococci, and staphylococci (4, 5, 10, 21), which are also present at high levels in colostrum and breast milk, whereas bifidobacteria are detected at very low levels (14). In contrast, from day six to one month old, the microbiota becomes stable and dominated by bifidobacteria (4). *Bifidobacterium* species represent one of the main bacterial groups in the infant gut, particularly in breastfed infants (4, 7, 18), thereby linking the presence of high levels of bifidobacteria with the timely and appropriate development and maturation of the immune system (8, 20). However, the rapid establishment of *Bifidobacterium* in this environment is a challenge because anoxic conditions are not ensured in the infant gut during the first days following delivery. The exposure of bifidobacteria to oxygen causes oxidative stress due to the accumulation of reactive oxygen species, mainly hydrogen peroxide (H_2_O_2_), which may lead to cell death (11, 16, 19). Catalase represents the main line of defense against H_2_O_2_ in many kinds of bacteria. The production of catalase has been reported in *Bifidobacterium indicum* and *Bifidobacterium asteroides* asteroids, but is absent in most bifidobacteria (2). According to He *et al.*(9), the acquired tolerance to oxidative stress of *Bifidobacterium longum* 105-A via the heterologous expression of a catalase gene revealed that the accumulation of H_2_O_2_ was a primary factor for the inhibition of growth by bifidobacteria. Previous studies reported that the addition of catalase improved the aerobic growth of bifidobacteria (3, 11). Catalase-positive *E. coli* has been shown to cross-protect adjacent catalase-deficient (*ΔkatE*) mutant *E. coli* K-12 (12) colonies against a H_2_O_2_ challenge (13).

The aim of the present study was to investigate the tolerance of bifidobacteria strains isolated from infant gut and breast milk to oxygen and determine if catalase-positive bacteria present in human niches exhibited the ability to cross-protect adjacent bifidobacteria from oxidative stress, thereby allowing their survival and multiplication under environmental conditions with exposure to oxygen.

Eighty-five strains of bifidobacteria previously isolated from the infant gut and human breast milk (17) were investigated in the present study (Fig. 1). Strains were grown in Reinforced Clostridial agar (RCA; Difco Laboratories, Sparks, MD, USA) for 48 h at 37°C under anaerobic conditions (AnaeroGen; Oxoid, Basingstoke, Hampshire, UK). Catalase-positive *S. epidermidis* INIA P190, *E. coli* INIA P114, and *E. coli* K-12, which were obtained from the National Institute for Agricultural and Food Research and Technology (INIA) Culture Collection, were grown in Brain Heart Infusion broth (BHI; Merk, Darmstadt, Germany) for 18 h at 37°C. A catalase-deficient mutant (*ΔkatE*) strain of *E. coli* K-12 (JW1721-1) (1), which showed no assayable catalase activity, was provided by the *E. coli* Genetic Stock Center (Yale University, CT, USA). Stock cultures were kept frozen at −80°C in the appropriate broth containing 10% (v/v) glycerol. All strains were subcultured twice before being used in experiments.

Suspensions (at approximately 7 log CFU mL^−1^) of bifidobacteria were prepared in duplicate by collecting cells from RCA plates and resuspending them in 2 mL of BHI broth to test their tolerance to oxidative stress. Bacterial suspensions were streaked onto BHI agar plates with or without catalase (Catalase type C-10; Sigma-Aldrich, St. Louis, MO, USA) at 0.2 g L^−1^, equivalent to 520 AU mL^−1^ agar, and cultured for 48 h at 37°C under microaerobic conditions (GENbox microaer; bioMérieux, Marcy L’Etoile, France) as well as anaerobic and aerobic conditions. Based on their behavior under microaerobic conditions, bifidobacteria were classified into three classes: unable to grow (class 1), able to grow (class 2), and only able to grow in the presence of catalase (class 3).

Representative bifidobacteria strains from the three classes previously described were investigated in mixed culture assays with the breast fed infant gut catalase-positive isolates *S. epidermidis* INIA P190 and *E. coli* INIA P114, and with *E. coli* K-12 and its catalase-deficient (*ΔkatE*) mutant *E. coli* JW1721-1. Mixed cultures in pairs combining bifidobacteria (at approximately 7 log CFU mL^−1^) with each of the catalase-positive strains (at approximately 3 log CFU mL^−1^) were spread onto BHI agar plates and cultured under micro-aerobic conditions for 48 h at 37°C. The identity of presumptive bifidobacteria colonies was confirmed by observing the characteristic bifid morphology with phase-contrast microscopy and by colony PCR with specific primers and conditions previously described (17).

All the bifidobacteria tested grew well under anaerobic conditions and were unable to grow under aerobic conditions. According to their behavior under microaerobic conditions, bifidobacteria were classified into three classes (Fig. 1). A total of 31% of the strains were not able to grow under microaerobic conditions even when catalase was present (class 1). This group included *Bifidobacterium adolescentis* (100%), *Bifidobacterium catenulatum* (80%), and *Bifidobacterium pseudocatenulatum* (80%). Only 10% of all the strains tested tolerated microaerobic conditions and formed proper colonies (class 2). This group encompassed some strains belonging to *Bifidobacterium bifidum* (10%), *Bifidobacterium breve* (20%), and *B. longum* subsp. *infantis* (50%). When catalase was added, 59% of the strains tested were able to grow under microaerobic conditions (class 3). This group included strains of all the species tested, except for *B. adolescentis*. Kawasaki *et al.*(11) previously reported that the growth of two strains of bifidobacteria, which was inhibited due to the accumulation of H_2_O_2_, was restored by the addition of catalase to the medium. The bifidobacteria species described in the literature as being common in the first days of life in the infant gut (18) were those exhibiting higher tolerance to oxygen in the present study (*B. bifidum*, *B. breve*, and *B. longum* subsp. *infantis*), and mainly belonged to classes 2 and 3. These results suggested that some bifidobacteria remained in the microaerobic parts of the intestine and mammary ducts as long as H_2_O_2_ produced by that species was neutralized. Therefore, we investigated the potential cross-protection of catalase-positive bacteria, typically present in the neonate gut or in human breast milk (10), against H_2_O_2_ produced by bifidobacteria in the presence of low oxygen levels.

Three representative strains from each of the classes described above, *B. longum* subsp. *longum* INIA P721, *B. adolescentis* INIA P879, and *B. pseudocatenulatum* INIA P888 from class 1; *B. bifidum* INIA P671, *B. breve* INIA P714, and *B. longum* subsp. *infantis* INIA P718 from class 2; and *B. breve* INIA P244, *B. bifidum* INIA P745, and *B. longum* subsp. *longum* INIA P748 from class 3, were studied in mixed culture assays with the catalase-positive isolates in solid media. The bifidobacteria from class 1 did not grow in the presence of catalase-positive bacteria, while those from class 2 grew together with catalase-positive strains over all the agar plate. The strains belonging to class 3 showed small colonies adjacent to the catalase-positive colonies. Typical images of strains from the three classes growing on BHI agar in a mixed culture with *S. epidermidis* INIA P190, *E. coli* INIA P114, or *E. coli* K-12 under microaerobic conditions are shown in Fig. 2 and Fig. S1. The cells from these small colonies showed the typical bifid morphology under phase contrast microscopy (Fig. S2) and were identified as *Bifidobacterium* sp. by PCR (data not shown). In contrast to the parental strain *E. coli* K-12, no bifidobacteria colonies belonging to class 3 were observed adjacent to the catalase-deficient (*ΔkatE*) *E. coli* mutant during the mixed culture assays carried out with the selected strains, indicating that catalase played a key role in this event (Fig. 2f). According to these results, gut catalase-positive bacteria protected neigh-boring catalase-deficient bifidobacteria against oxidative stress, thereby assisting in their establishment in the infant gastrointestinal tract or other non-anaerobic human niches such as the mammary gland. The gut microbiota is a complex ecosystem in which microorganisms live in a balanced relationship with other species that often occur in dense biofilms. Ma and Eaton (13) supported bacterial catalase protecting high-density or colonial, but not individual *E. coli* against environmental H_2_O_2_. This group protection may extend to neighboring catalase-negative *E. coli*.

In conclusion, this study shows differences in the tolerance of bifidobacterial species to oxidative stress, pointing out the competitive advantage of some strains of *B. bifidum*, *B. breve*, *B. longum* subsp. *infantis*, and *B. longum* subsp. *longum* in the colonization process of the infant gut microbiota, being the cross-protection of gut catalase-positive bacteria a possible mechanism towards the hydrogen peroxide generated by *Bifidobacterium* species.

## Supplementary Information



## Figures and Tables

**Fig. 1:**
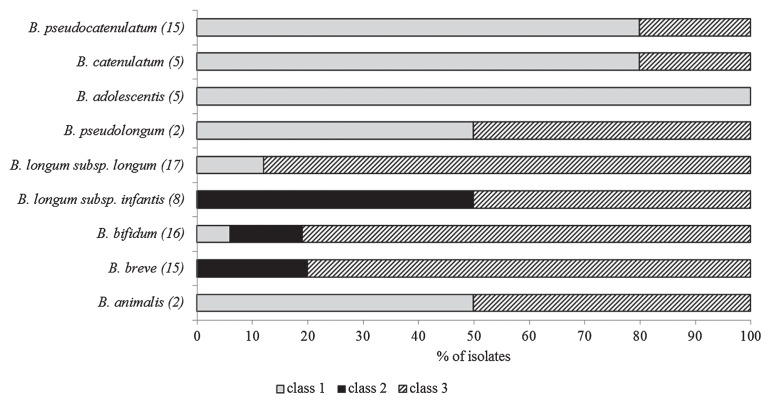
Distribution and number (in brackets) of bifidobacteria species, according to their ability to grow under microaerobic conditions in the presence/absence of catalase. Class 1: No growth; Class 2: Growth; Class 3: Growth only in the presence of catalase (520 AU mL^−1^).

**Fig. 2:**
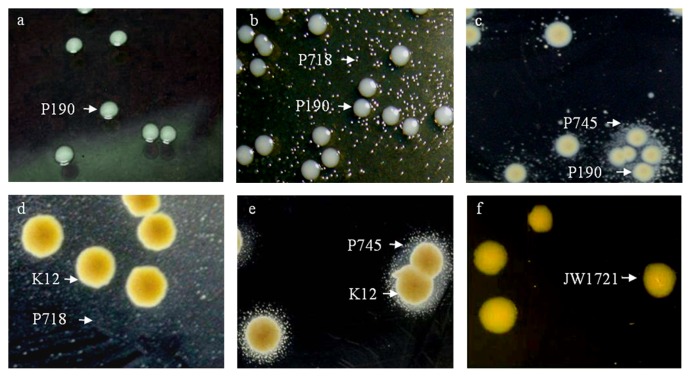
Growth under microaerobic conditions of *Staphylococcus epidermidis* INIA P190 in a mixed culture with: (a) class 1 *Bifidobacterium adolescentis* INIA P879; (b) class 2 *Bifidobacterium longum* INIA P718; or (c) class 3 *Bifidobacterium bifidum* INIA P745; *Escherichia coli* K-12 in a mixed culture with: (d) *B. longum* INIA P718 or (e) *B. bifidum* INIA P745; and (f) catalase-deficient mutant *E. coli* JW1721-1 in a mixed culture with *B. bifidum* INIA P745. Representative colonies are indicated by arrows with the strain name.

## References

[1] Baba T, Ara T, Hasegawa M, Takai Y, Okumura Y, Baba M, Datsenko KA, Tomita M, Wanner BL, Mori H (2006). Construction of *Escherichia coli* K-12 in-frame, single-gene knockout mutants: the Keio collection. Mol Syst Biol.

[2] Biavati B, Mattarelli P, Falkow S, Rosenberg E, Schleifer KH, Stackebrandt E, Dworkin M (2001). The family Bifidobacteriaceae. The Prokaryotes: An Evolving Electronic Resource for the Microbiological Community.

[3] De Vries W, Stouthamer AH (1969). Factors determining the degree of anaerobiosis of *Bifidobacterium* strains. Arch Mikrobiol.

[4] Fanaro S, Chierici R, Guerrini P, Vigi V (2003). Intestinal microflora in early infancy: composition and development. Acta Pediatr Suppl.

[5] Favier CF, de Vos WM, Akkermans ADL (2003). Development of bacterial communities in faeces of newborn babies. Anaerobe.

[6] Grönlund MM, Grzeskowiak L, Isolauri E, Salminen S (2011). Influence of mother’s intestinal microbiota on gut colonization in the infant. Gut Microbes.

[7] Gueimonde M, Laitinen K, Salminen S, Isolauri E (2007). Breast milk: a source of bifidobacteria for infant gut development and maturation?. Neonatology.

[8] Hart AL, Lammers K, Brigidi P, Vitali B, Rizzello F, Gionchetti P, Campieri M, Kamm MA, Knight SC, Stagg AJ (2004). Modulation of human dendritic cell phenotype and function by probiotic bacteria. Gut.

[9] He J, Sakaguchi K, Suzuki T (2012). Acquired tolerance to oxidative stress in *Bifidobacterium longum* 105-A via expression of a catalase gene. Appl Environ Microbiol.

[10] Jost T, Lacroix C, Chassard C (2012). New insights in gut microbiota establishment in healthy breast fed neonates. PLOS ONE.

[11] Kawasaki S, Mimura T, Satoh T, Takeda K, Niimura Y (2006). Response of the microaerophilic *Bifidobacterium* species, *B. boum* and *B. thermophilum*, to oxygen. Appl Environ Microbiol.

[12] Loewen PC (1984). Isolation of catalase-deficient *Escherichia coli* mutants and genetic mapping of *katE*, a locus that affects catalase activity. J Bacteriol.

[13] Ma M, Eaton W (1992). Multicellular oxidant defense in unicellular organisms. Proc Natl Acad Sci USA.

[14] Martín R, Jiménez E, Heilig H, Fernández L, Marín ML, Zoetendal EG, Rodríguez JM (2009). Isolation of bifidobacteria from breast milk and assessment of the bifidobacterial population by PCR-denaturing gradient gel electrophoresis and quantitative real-time PCR. Appl Environ Microbiol.

[15] Mikami K, Takahashi H, Kimura M, Isozaki M, Izuchi K, Shibata R, Sudo N, Matsumoto H, Koga Y (2009). Influence of maternal bifidobacteria on the establishment of bifidobacteria colonizing the gut in infants. Pediatr Res.

[16] Mozzetti V, Grattepanche F, Moine D, Berger B, Rezzonico E, Meile L, Arigoni F, Lacroix C (2010). New method for selection of hydrogen peroxide adapted bifidobacteria cells using continuous culture and immobilized cell technology. Microb Cell Fact.

[17] Rodríguez E, Arqués JL, Rodríguez R, Peirotén A, Landete JM, Medina M (2012). Antimicrobial properties of probiotic strains isolated from breast-fed infants. J Funct Foods.

[18] Roger LC, Costabile A, Holland DT, Hoyles L, McCartney AL (2010). Examination of faecal *Bifidobacterium* populations in breast- and formula-fed infants during the first 18 months of life. Microbiology.

[19] Talwalkar A, Kailasapathy K (2004). The role of oxygen in the viability of probiotic bacteria with reference to *L. acidophilus* and *Bifidobacterium* spp. Curr Issues Intest Microbiol.

[20] Turroni F, Peano C, Pass DA (2012). Diversity of bifidobacteria within the infant gut microbiota. PLOS ONE.

[21] Yoshioka H, Iseki K, Fujita K (1983). Development and differences of intestinal flora in the neonatal period in breast-fed and bottle fed infants. Pediatrics.

