# Supplementary material for: Gut Catalase-Positive Bacteria Cross-Protect Adjacent Bifidobacteria from Oxidative Stress
Source: Microbes Environ. 2015 Jun 4;30(3):270–2. doi: 10.1264/jsme2.ME15025 (PMC4567566; doi:10.1264/jsme2.ME15025)

Fig. S1. Growth under microaerobic conditions of *Staphylococcus epidermidis* INIA P190 in mixed culture with: (a) class 2 *Bifidobacterium breve* INIA P714 or (b) class 3 *B. longum* INIA P748; *Escherichia coli* K12 in mixed culture with: (c) *B. breve* INIA P714 (d), *B. longum* INIA P748 or (e) class 3 *B. breve* INIA P244; and *E. coli* INIA P114 in mixed culture with: (f) class 2 *Bifidobacterium bifidum* INIA P671, (g) class 3 *B. bifidum* INIA P745 or (h) *B. longum* INIA P748.

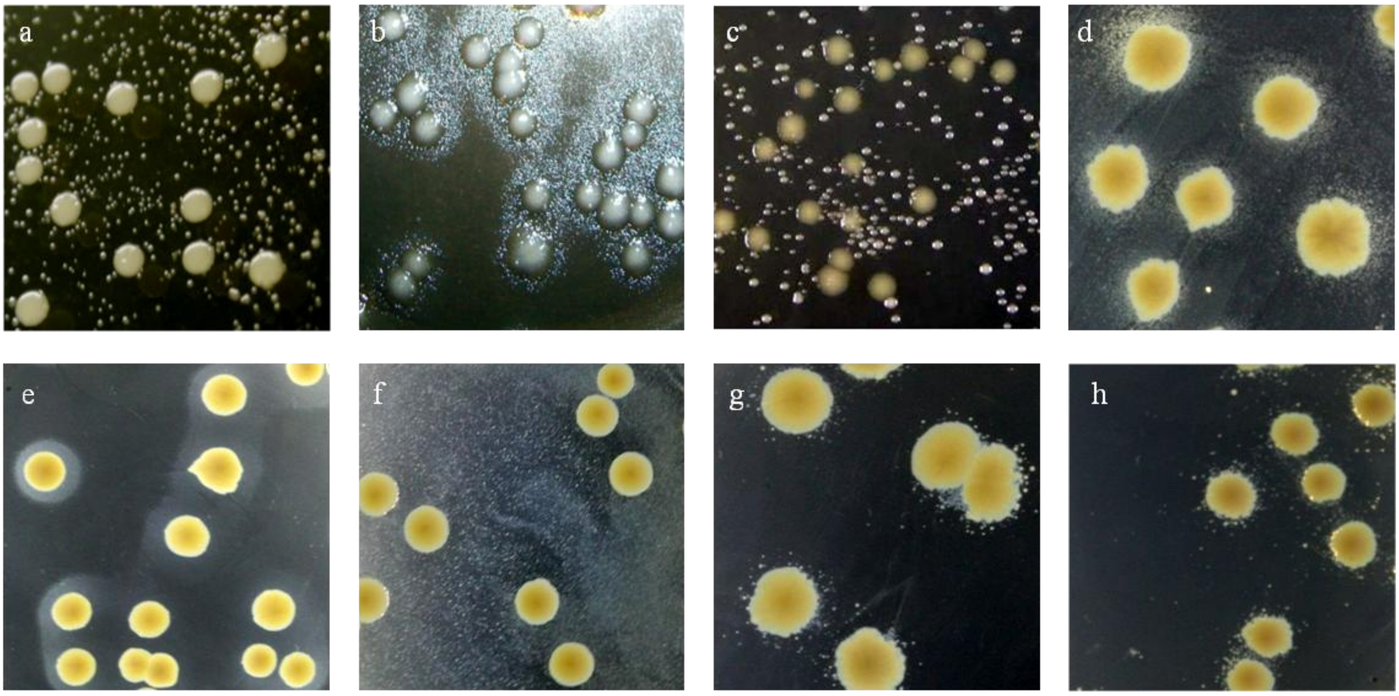

Fig. S2. Typical phenotypes by phase contrast microscopy from colonies from mixed culture assays: (a) *B. bifidum* INIA P745 (see Fig. 2c, 2e and S1g); and (b) *B. longum* INIA P748 (see Fig. S1b, S1d and S1h).

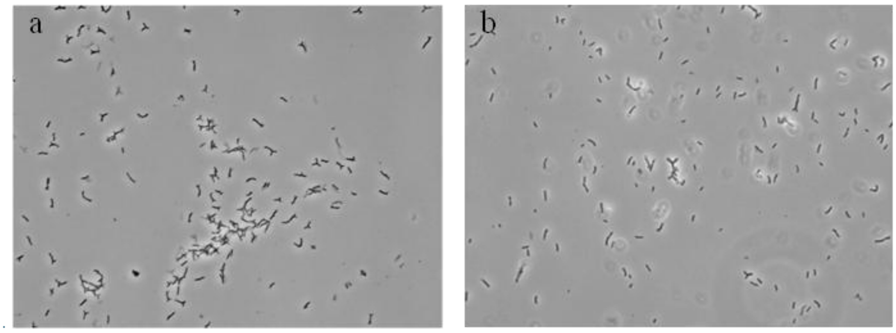

Supplement: Supplementary file 1 [file 30_270_s1.pdf]
